# Task specific effects of fatigue on lower limb muscle synergies during single and double leg drop jumps in female athletes

**DOI:** 10.3389/fphys.2026.1897091

**Published:** 2026-07-10

**Authors:** Jiawei Zheng, Junfeng Teng, Kunpeng Wang, Xikai Lin

**Affiliations:** 1 School of Sports Medicine, Wuhan Sports University, Wuhan, China; 2 Pain management and Tuina Department, Beijing University of Chinese Medicine Shenzhen Hospital (Longgang), Shenzhen, Longgang, China

**Keywords:** drop jump, electromyography, fatigue, muscle synergy, non-negative matrix factorization

## Abstract

**Background:**

Drop jump (DJ) tasks are widely used to assess lower-limb neuromuscular function and injury risk. Fatigue is a key factor elevating injury risk, yet its effects on neuromuscular control during single-leg drop jumps (SLDJ) and double-leg drop jumps (DLDJ), and whether these effects differ by task type, remain poorly understood.

**Methods:**

Fifteen female athletes performed SLDJ and DLDJ before and after a standardized fatigue protocol. Surface electromyography signals from nine lower-limb muscles were collected. Muscle synergies were extracted via non-negative matrix factorization, and synergy similarity was assessed using scalar product and Pearson correlation coefficients. Linear mixed-effects models examined differences in muscle contributions and temporal activation features.

**Results:**

Three muscle synergy modules were consistently identified across both tasks and fatigue conditions, with high inter-module similarity (scalar product and Pearson r > 0.80). Synergy structure remained stable regardless of task or fatigue, while task type and fatigue selectively modulated specific muscle contributions. Notably, vastus lateralis contribution showed opposing fatigue-related trends between tasks, and lateral gastrocnemius contribution decreased significantly after fatigue in SLDJ only.

**Conclusions:**

The central nervous system maintains a stable modular control strategy across DJ tasks and fatigue conditions. Adaptations to fatigue manifest primarily through task-specific modulation of intra-module muscle weights rather than restructuring of synergy organization. SLDJ demonstrated greater neuromuscular sensitivity to fatigue than DLDJ, indicating that single-leg landing tasks may be more susceptible to fatigue-induced alterations in neuromuscular control. These findings inform neuromuscular screening and targeted training strategies for female athletes.

## 1 Introduction

Drop jump (DJ) tasks require participants to rapidly decelerate after landing from a given height and immediately perform an explosive vertical takeoff. Because DJ involves rapid eccentric braking and substantial ground impact forces, it is widely used in sports science to assess athletes’ stretch-shortening cycle (SSC) efficiency and mechanisms of lower-limb injury (Kotsifaki et al., 2023; Pedley et al., 2025; Zhang et al., 2025). According to the landing strategy, DJ tasks are commonly classified as single-leg drop jumps (SLDJ) and double-leg drop jumps (DLDJ) (Taylor et al., 2016). Previous studies have shown that, compared with DLDJ, SLDJ often produces greater ground reaction forces (GRF) and smaller knee flexion angles, suggesting higher injury risk and greater demands on the neuromuscular system (Pappas et al., 2007; Yeow et al., 2010; Ch et al., 2011). However, existing studies have mainly explained these differences from kinematic and kinetic perspectives, while the underlying neuromuscular control mechanisms remain insufficiently explored. Clarifying the neuromuscular control differences between SLDJ and DLDJ may help explain their distinct biomechanical characteristics and provide evidence for athlete screening and targeted training.

Fatigue is also widely considered an important factor that increases the risk of sport-related injury (Zou et al., 2024; Liu et al., 2025). Epidemiological evidence has shown that the incidence of anterior cruciate ligament (ACL) injury increases significantly during the later stages of games as fatigue accumulates (IRR = 2.40; 95% CI = 1.15–5.02) (Anderson et al., 2019). Laboratory studies further indicate that fatigue alters hip, knee, and ankle biomechanics during DJ tasks (Liu et al., 2025; Prieske et al., 2017; Fort-Vanmeerhaeghe et al., 2023; Kamitani et al., 2023). A recent meta-analysis reported that fatigue-induced changes in dynamic knee valgus angle are inconsistent across different landing tasks, suggesting that the effects of fatigue on neuromuscular control may be modulated by jump or landing type (Anderson et al., 2019). However, although many studies have investigated the effects of fatigue on drop jump tasks, existing evidence remains largely focused on kinematic and kinetic characteristics. How fatigue influences the coordinated control of lower-limb muscles by the central nervous system (CNS) during drop jump tasks, and whether this influence differs across DJ types, remains insufficiently understood.

In recent years, muscle synergy analysis has been widely used in sports science. This approach assumes that the central nervous system (CNS) simplifies motor control by organizing multiple muscles into a small number of synergy modules, rather than controlling each muscle independently (d’Avella et al., 2003; Bizzi et al., 2008; Hirashima and Oya, 2016). Based on non-negative matrix factorization, muscle synergy analysis decomposes multi-channel EMG signals into muscle-weighting patterns and temporal activation patterns, thereby reflecting the neuromuscular control strategy underlying a movement task (Shourijeh et al., 2016; Turpin et al., 2021). Previous studies on repetitive tasks such as running and upper-limb pointing have shown that fatigue generally does not markedly alter the overall synergy structure, but mainly adjusts the contribution weights or activation features of specific muscles (Hajiloo et al., 2020; Thomas et al., 2023; Chen et al., 2024; Xu et al., 2025). However, whether this pattern also applies to rapid explosive tasks such as DJ, and whether synergy patterns differ between DJ types or fatigue conditions, remains unclear.

Therefore, this study used muscle synergy analysis based on NMF to achieve two objectives: first, to compare the characteristics of lower-limb muscle synergy patterns during SLDJ and DLDJ in female athletes; and second, to investigate the effects of fatigue on lower-limb muscle synergy patterns during SLDJ and DLDJ, and to determine whether these effects differ according to DJ type. We hypothesized that, first, SLDJ and DLDJ would share a similar overall synergy structure but differ in specific muscle contributions and activation features; and second, fatigue would alter lower-limb muscle synergy patterns during DJ tasks, and these changes would differ between DJ types.

## 2 Methods

### 2.1 Participants

Referring to similar previous studies (Li and He, n.d.), an *a priori* power analysis was conducted using G*Power. With an effect size of f = 0.35, α = 0.05, and β = 0.08, the calculated minimum required sample size was 13 participants. A total of 15 female athletes were recruited from Wuhan Sports University for this study (age: 20.9 ± 2.2 years; height: 167.8 ± 5.8 cm; body mass: 57.9 ± 7.2 kg; training experience: 8.0 ± 2.6 years; weekly training duration: 9.3 ± 6.4 h). All participants were highly trained basketball or volleyball athletes, holding at least a National Level II athlete certification in China. Limb dominance was determined using the Waterloo Footedness Questionnaire-Revised, and all participants were identified as right-leg dominant (van Melick et al., 2017). In addition, none of the participants had a history of lower-limb injury within the six months prior to testing.

The study protocol was approved by the Ethics Committee of Wuhan Sports University (Approval No. 2025171) and conducted in accordance with the Declaration of Helsinki. Prior to participation, all subjects were fully informed about the experimental procedures and potential risks, and written informed consent was obtained from each participant.

### 2.2 Fatigue protocol

A combined protocol consisting of vertical jumps and shuttle sprints was used to induce fatigue (Chappell et al., 2005). Specifically, participants initiated each cycle from a squatting position and performed five consecutive vertical jumps, with each jump required to reach at least 115% of their individual maximal vertical reach height. Immediately following the jumps, participants completed a 15-m shuttle sprint. This sequence was repeated continuously until the fatigue criteria were met. Fatigue was determined using a combination of physiological and perceptual measures. Heart rate was continuously monitored using a Polar wireless heart rate monitor (Polar Electro Oy, Kempele, Finland), with real-time heart rate data displayed and recorded via the Polar Beat smartphone application. Fatigue was considered achieved when the participant’s heart rate reached at least 85% of their age-predicted maximum (220 − age), in conjunction with a rating of perceived exertion (RPE) score ≥17 on the Borg 6–20 scale (Borg et al., 2010; Stojanović et al., 2018). Heart rate and RPE were periodically checked to ensure participants remained above the predefined fatigue thresholds throughout the post-fatigue trials. Changes in jump performance variables were also used to provide functional evidence supporting the effectiveness of the fatigue protocol (Franceschi et al., 2023).

### 2.3 Experimental procedure

During testing, participants wore standardized tight-fitting pants provided by the laboratory along with their own athletic shoes. After completing a 10-minute standardized warm-up on a treadmill, participants proceeded to the formal testing session.

Surface electromyography (EMG) electrodes (Ag/AgCl, disposable) were placed on lower limb in accordance with SENIAM guidelines. The muscles included the gluteus medius (Gmed), rectus femoris (RF), vastus medialis obliquus (VMO), vastus lateralis (VL), biceps femoris (BF), semitendinosus (ST), tibialis anterior (TA), peroneus longus (PL), and lateral gastrocnemius (LGM), with electrodes aligned parallel to the muscle fiber orientation (Hermens et al., 2000). Prior to electrode placement, the skin was prepared by shaving, abrasion, and cleansing with alcohol to minimize impedance. EMG signals were recorded unilaterally from the dominant limb during both SLDJ and DLDJ. The same limb was examined across all task and fatigue conditions to provide a standardized within-participant comparison and to minimize variability associated with limb dominance.

**Figure 1:**
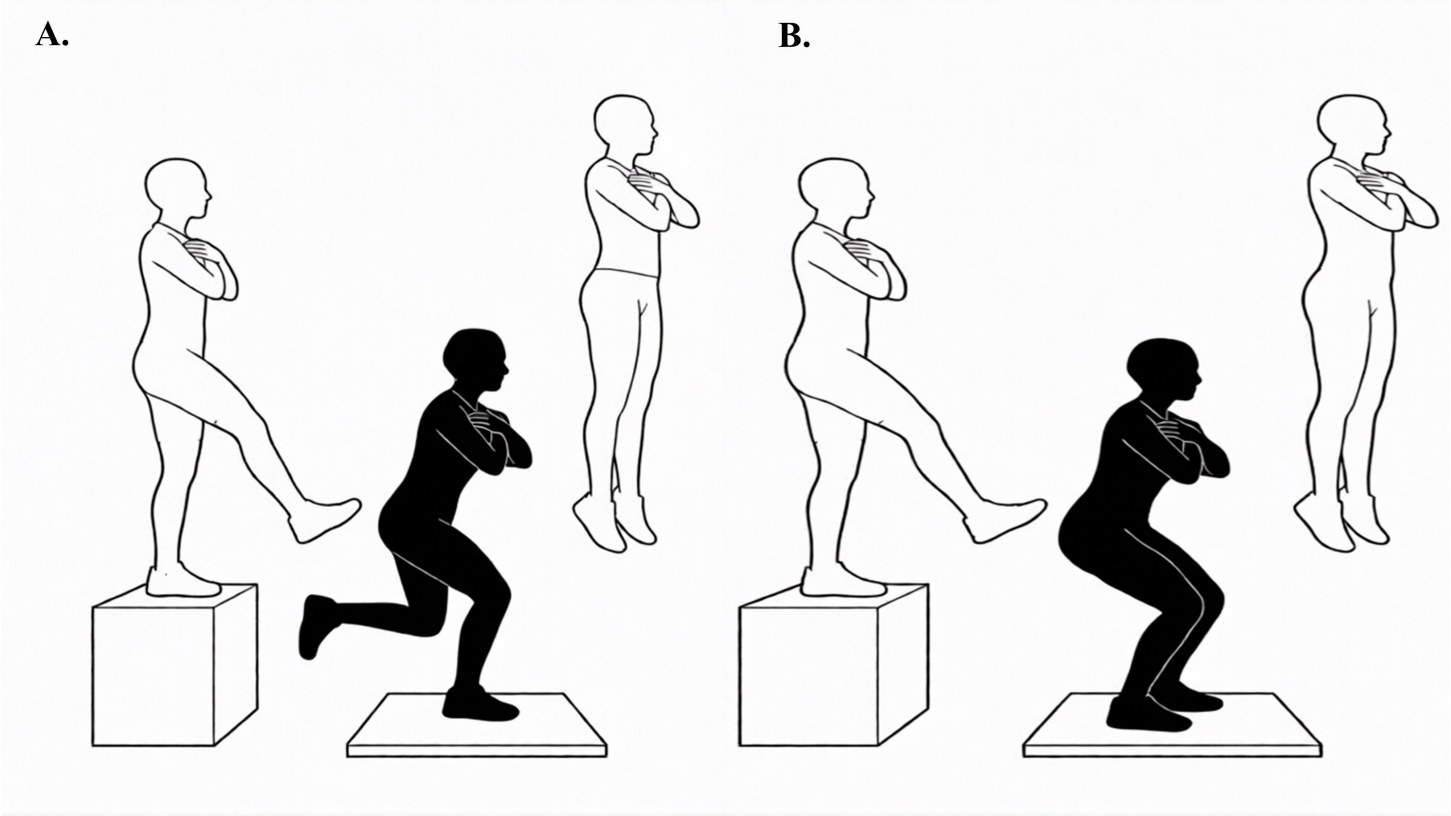
Diagram of the movement. **(A)** Single-leg drop jump. **(B)** Double-leg drop jump.

Following electrode placement, participants performed SLDJ and DLDJ tasks in a randomized order. During testing, participants were instructed to keep their hands crossed over the chest and step off a 30-cm platform. They landed on a force plate positioned at a height equivalent to 50% of the platform height and immediately performed a maximal vertical jump using either one or both legs (**Figure 1**). Three valid trials were collected for each condition both before and after fatigue. During post-fatigue testing, participants were required to perform five consecutive vertical jumps between every two trials to maintain the fatigued state (Chappell et al., 2005). GRF data were recorded using a Kistler force plate (1200 Hz), and EMG signals were synchronously collected using a Noraxon wireless EMG system (2000 Hz).

### 2.4 Data processing

All kinetic and EMG data were processed using MATLAB R2024a (MathWorks, USA). GRF data in the vertical direction were filtered using a zero-lag fourth-order Butterworth low-pass filter with a cutoff frequency of 50 Hz. A threshold of 10 N was used to determine initial contact (IC) and toe-off (TO) events (Wilder et al., 2021). Subsequently, jump height(JH), reactive strength index(RSI), and ground contact time (GTC) were calculated to objectively evaluate the effectiveness of the fatigue protocol (Fitzpatrick et al., 2019; Franceschi et al., 2023). JH was computed using the flight time method, and RSI was calculated as jump height divided by GCT (Merrigan et al., 2022). For EMG data, the analysis window was defined from IC to TO during the drop jump task. Signal preprocessing included the following steps: (1) Raw EMG signals were band-pass filtered (20–500 Hz) using a zero-lag fourth-order Butterworth filter, followed by full-wave rectification and low-pass filtering at 6 Hz to obtain the linear envelope. (2) Given that EMG normalization methods can influence the number, structure, and temporal features of extracted muscle synergies, inter-trial maximum normalization was used in this study based on previous methodological recommendations (Ortega-Auriol et al., 2025). For each muscle, the normalization value was defined as the mean EMG amplitude within a 100 ms window centered around the peak activation across trials, which was intended to improve the stability of synergy extraction and reduce the influence of transient signal fluctuations. (3) Time normalization was conducted using interpolation to resample each trial to 101 data points, corresponding to 0–100% of the stance phase (from IC to TO). After preprocessing, EMG data from nine muscles across three trials (101 data points per trial) were concatenated to construct a 9 × 303 matrix for each condition, which was used as input for subsequent muscle synergy extraction and analysis.

### 2.5 Muscle synergy extraction

Muscle synergies were extracted from the preprocessed EMG data using non-negative matrix factorization (NMF) based on a multiplicative update algorithm (d’Avella and Bizzi, 2005). The EMG matrix was factorized as follows:


$$M_{t\times m}\approx H_{t\times n}\cdot W_{n\times m}$$

where *M* represents the original EMG matrix, *t* is the number of time samples, and *m* is the number of muscles. Matrix *H* denotes the motor primitives, reflecting the time-varying activation profiles of each synergy, while *W* represents the motor modules, indicating the relative contribution of each muscle within a synergy. The variable *n* corresponds to the number of synergies.

To determine the optimal number of synergies, NMF was iteratively performed with *n* ranging from 1 to 9. The quality of reconstruction was evaluated using the coefficient of determination (*R*^2^), calculated as follows (d’Avella et al., 2006):


$$R^{2}=1-\frac{\sum_{i,j}{\left(M_{i,j}-M_{i,j}^{"}\right)}^{2}}{\sum_{i,j}{\left(M_{i,j}-\bar{M}\right)}^{2}}$$

where $M_{i,j}\;$ and $M_{i,j}^{"}\;$ represent the original and reconstructed EMG signals, respectively, and $\bar{M\;}$ is the mean value of the original matrix. For each predefined number of synergies, the NMF algorithm was repeated fifty times with random initializations. Convergence was assumed when the change in *R*^2^ over the last 20 iterations was less than 0.01%. The highest *R*^2^ value among the five runs was retained. The optimal number of synergies for each task condition was determined by cross-validating two complementary criteria (Herzog et al., 2025): (1) R²-knee-point criterion, identified as the first n at which the MSE of an iteratively fitted linear regression to the R² curve fell below 10^-^^4^; (2) A dual global–local threshold criterion, defined as the minimum n satisfying $R^{2}\geq 0.90$ and an incremental improvement $(R_{n+1}^{2}-R_{n}^{2})<0.05$.

To enable comparisons of muscle synergy patterns across fatigue conditions and drop jump tasks, k-means clustering was applied to the motor modules derived from pre-fatigue data for each task separately to identify representative synergy patterns. The number of clusters (*k*) was systematically varied from 2 to 5, with 50 random initializations performed for each *k* to avoid local minima. The optimal cluster number was determined by maximizing the global mean silhouette coefficient. Subsequently, motor modules extracted under post-fatigue conditions were matched to the pre-fatigue cluster centroids using Pearson correlation coefficients. A module was assigned to a given cluster if the correlation coefficient r≥0.60 (Xu et al., 2025). In cases where multiple modules from the same participant were assigned to the same cluster, only the module with the highest correlation coefficient was retained to ensure a one-to-one correspondence between participants and cluster representatives.

Muscles with median weighting coefficients greater than 0.30 within each motor module were defined as dominant contributors, allowing for the functional interpretation of each synergy (Liu et al., 2024). For temporal feature analysis, the center of activity (CoA) and full width at half maximum (FWHM) of the motor primitives were calculated to quantify the timing and duration of muscle activation, respectively (Ivanenko et al., 2005; Santuz et al., 2017).

### 2.6 Statistical analysis

A two-way repeated measure analysis of variance (ANOVA) was conducted to examine the main and interaction effects of fatigue condition and DJ type on the number of muscle synergies, reconstruction quality(***R***^2^), JH, RSI and GCT. To evaluate synergy similarity, both the scalar product and Pearson correlation coefficient were calculated for the motor modules and motor primitives. Similarity was assessed (1) between different DJ tasks under the same fatigue condition and (2) between fatigue conditions within the same task.

Due to potential splitting or merging of muscle synergy modules across experimental conditions, the number of observations within homologous modules may vary after clustering, resulting in an unbalanced dataset that violates the assumptions of repeated-measures ANOVA. Therefore, linear mixed-effects models(LMMs) were employed to analyze muscle contributions and temporal features, while accounting for subject-level random effects. For corresponding synergies with similarity coefficients greater than 0.80 (Saito et al., 2023), LMMs were constructed to further examine differences in the relative contribution of dominant muscles, as well as temporal features including the CoA and FWHM. In these models, fatigue condition, jump type, and their interaction were treated as fixed effects, while participant was included as a random effect to account for inter-individual variability (Herzog et al., 2025). Effect sizes for fixed effects were calculated as ηp² = t²/(t² + df), where t is the t statistic and df is the corresponding denominator degrees of freedom for each fixed effect (Selya et al., 2012). For both jump performance variables and muscle synergy-related outcomes, simple effects analyses were performed when a significant interaction effect was detected. Bonferroni correction was applied to adjust for multiple comparisons. The level of statistical significance was set at α = 0.05 for all analyses. Effect sizes for main and interaction effects were reported as ηp², with values of 0.01, 0.06, and 0.14 interpreted as small, medium, and large effects, respectively. Effect sizes for pairwise comparisons were reported as Cohen’s d, with values of 0.20, 0.50, and 0.80 interpreted as small, medium, and large effects, respectively. All statistical analyses were performed using Python 3.13 in PyCharm Community Edition.

## 3 Results

To improve readability, statistically significant findings are reported in detail in the main text, whereas non-significant results are briefly summarized in the Results section and reported in full in the **Supplementary Materials**.

### 3.1 Jump performance

A significant interaction was observed for JH (F = 5.08, P = 0.04, ηp²=0.26, 95%CI=0.00, 0.03). Simple effects analysis showed that, during SLDJ, JH significantly decreased from 0.126 ± 0.040 m before fatigue to 0.102 ± 0.023 m after fatigue (t = 3.39, p < 0.01, d = 0.87, 95% CI = 0.01, 0.04), whereas no significant fatigue-related change was observed during DLDJ (t=1.09, p =0.29, d=0.28, 95%CI=-0.001, 0.02). In addition, JH was significantly greater during DLDJ than SLDJ both pre-fatigue (t=-8.42, p<0.01, d=-2.17, 95%CI=-0.09, -0.05) and post-fatigue(t=-9.35, p<0.01, d=-2.41, 95%CI=-0.11, -0.07). Significant main effects of fatigue and jump type were found for RSI and GCT. RSI decreased post-fatigue (F = 24.76, p <0.01, ηp²=0.63, 95%CI=-0.14, -0.05), and was higher during DLDJ than SLDJ (F = 85.75, p <0.01, ηp²=0.86, 95%CI=0.29, 0.46). GCT increased after fatigue (F = 8.30, p =0.01, ηp²=0.37, 95%CI=0.01, 0.03), and was longer during SLDJ than DLDJ across both conditions (F = 8.21, p =0.01, ηp²=0.37, 95%CI=-0.05, -0.01).

**Figure 2:**
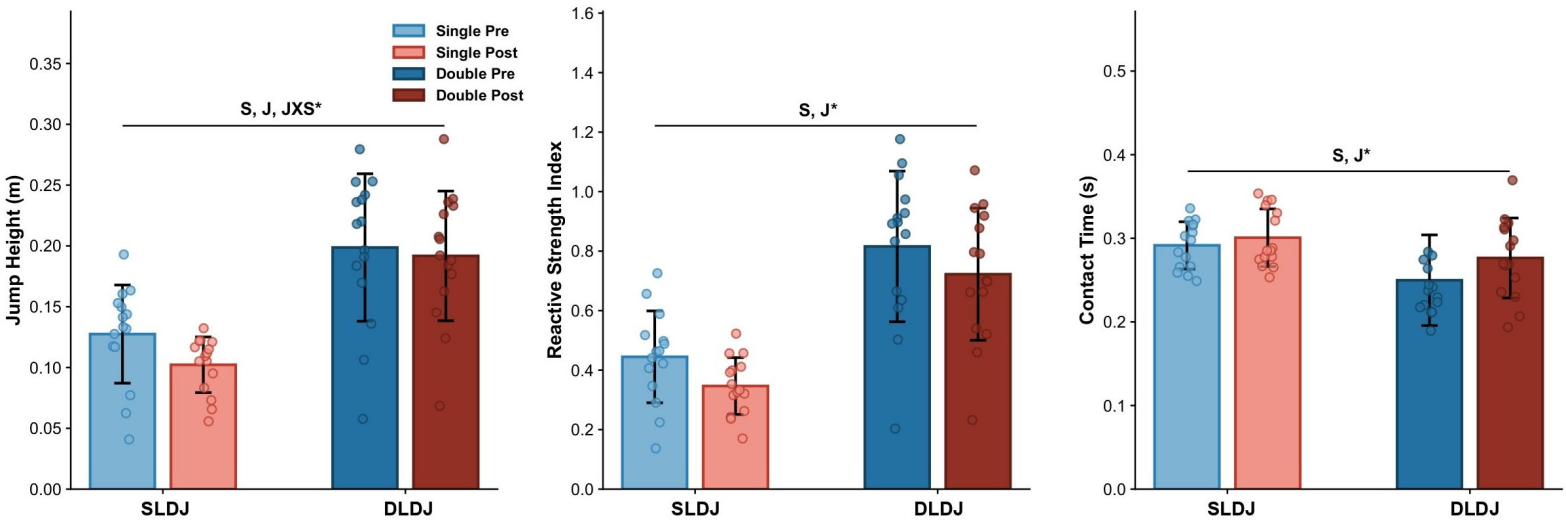
Jump performance; J* indicates a significant main effect of jump type; S* indicates a significant main effect of fatigue; S×J* indicates a significant interaction effect between fatigue and jump type.

**Figure 2** shows the JH, RSI and GCT during SLDJ and DLDJ pre- and post-fatigue. A significant interaction was observed for JH (F = 5.08, P = 0.04, ηp²=0.26, 95%CI=0.00, 0.03). Simple effects analysis showed that, during SLDJ, JH significantly decreased from 0.126 ± 0.040 m before fatigue to 0.102 ± 0.023 m after fatigue (t = 3.39, p < 0.01, d = 0.87, 95% CI = 0.01, 0.04), whereas no significant fatigue-related change was observed during DLDJ (t=1.09, p =0.29, d=0.28, 95%CI=-0.001, 0.02). In addition, JH was significantly greater during DLDJ than SLDJ both pre-fatigue (t=-8.42, p<0.01, d=-2.17, 95%CI=-0.09, -0.05) and post-fatigue(t=-9.35, p<0.01, d=-2.41, 95%CI=-0.11, -0.07). Significant main effects of fatigue and jump type were found for RSI and GCT. RSI decreased post-fatigue (F = 24.76, p <0.01, ηp²=0.63, 95%CI=-0.14, -0.05), and was higher during DLDJ than SLDJ (F = 85.75, p <0.01, ηp²=0.86, 95%CI=0.29, 0.46). GCT increased after fatigue (F = 8.30, p =0.01, ηp²=0.37, 95%CI=0.01, 0.03), and was longer during SLDJ than DLDJ across both conditions (F = 8.21, p =0.01, ηp²=0.37, 95%CI=-0.05, -0.01).

### 3.2 Number of synergies and *R*^2^

**Figure 3:**
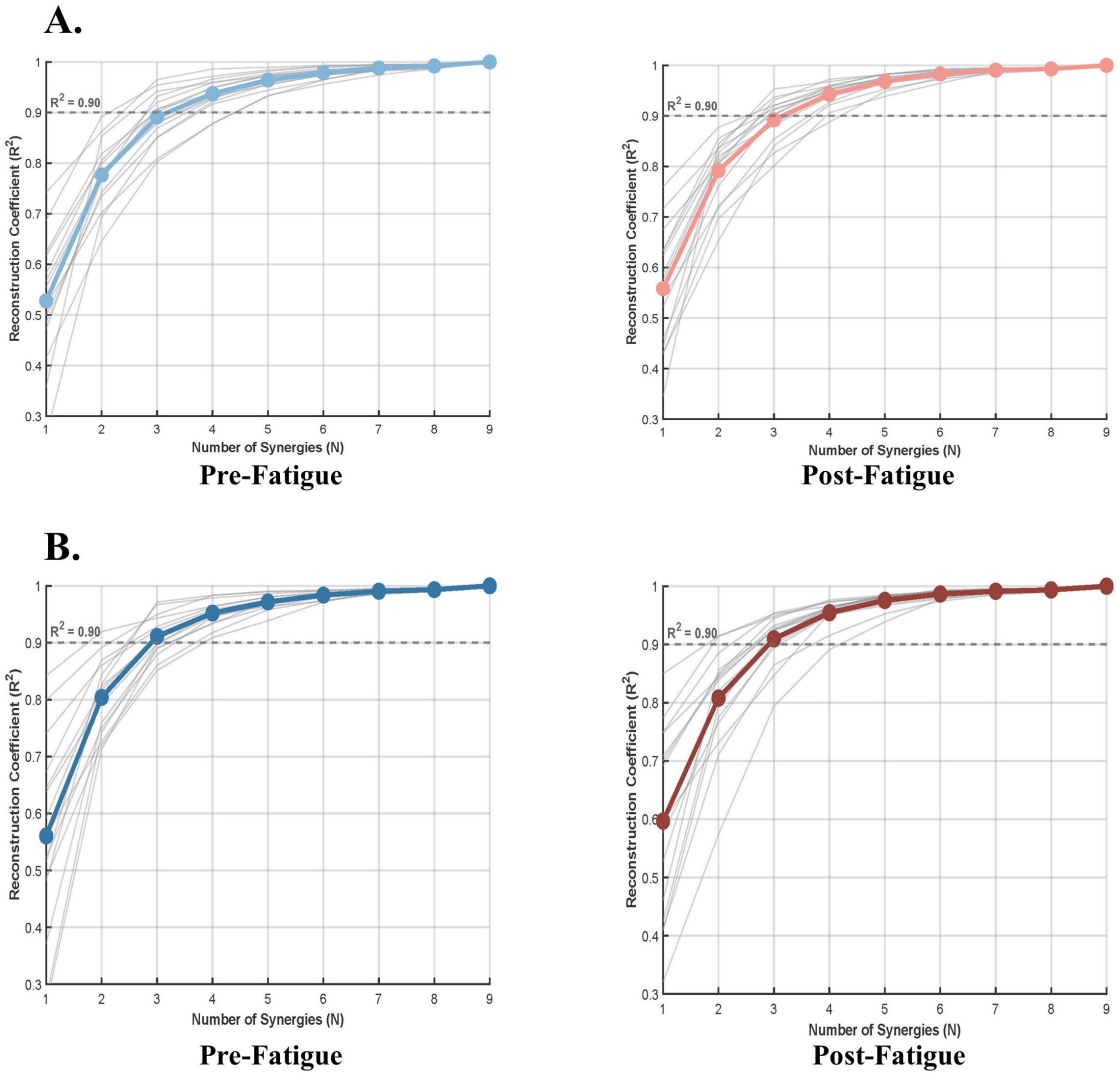
Number of synergies and *R*^2.^. **(A)** Single-leg drop jump. **(B)** Double-leg drop jump.

**Figure 3** shows the number of extracted muscle synergies and the *R*^2^ for SLDJ and DLDJ tasks under pre- and post-fatigue conditions. Specifically, for the SLDJ task, the number of synergies was 3.67 ± 0.72 before fatigue and 3.47 ± 0.64 after fatigue, with *R*^2^values of 0.93 ± 0.02 and 0.92 ± 0.02, respectively. For the DLDJ task, the number of synergies was 3.40 ± 0.63 pre-fatigue and 3.20 ± 0.77 post-fatigue, while the *R*^2^values were both 0.93 ± 0.02. Two-way repeated-measures ANOVA revealed no significant main effects of fatigue condition or drop jump type, nor any interaction effects, on either the number of muscle synergies or the *R*^2^(p>0.05).

### 3.3 Muscle synergy function and similarity

**Figure 4:**
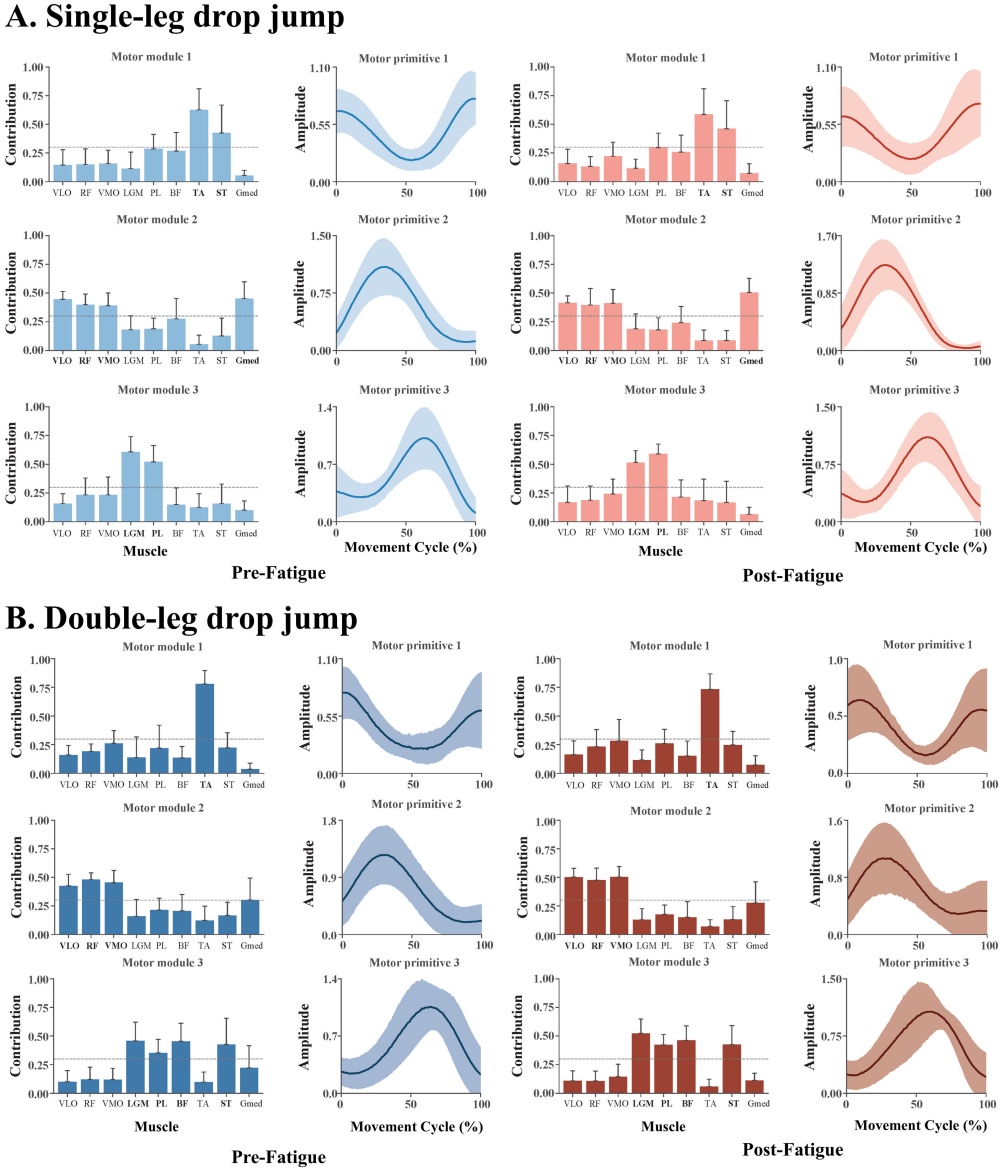
Muscle synergy function. The bar plots show the relative contribution of each muscle within a given synergy module, and muscles with weighting coefficients greater than 0.30 were identified as dominant contributors. The line plots illustrate the temporal activation profile of each synergy module throughout the drop jump phase. **(A)** SLDJ: Pre-Fatigue vs Post-Fatigue. **(B)** DLDJ: Pre-Fatigue vs Post-Fatigue. **(C)** Pre-Fatigue: SLDJ vs DLDJ. **(D)**Post-Fatigue: SLDJ vs DLDJ.

**Figure 4** shows the muscle synergy patterns identified through cluster analysis. Based on the silhouette coefficient, three clusters were determined as the optimal number of synergies for both SLDJ and DLDJ under pre-fatigue conditions. Synergy 1 exhibited peak activation around IC and TO. The dominant muscles were the TA and ST in SLDJ, whereas only the TA was identified as dominant in DLDJ. Synergy 2 was primarily active during approximately 25–40% of the movement cycle, corresponding to the landing and shock absorption phase. The dominant muscles included the VL, VMO, RF, and Gmed in SLDJ, and the VL, VMO, and RF in DLDJ. Synergy 3 was predominantly activated during approximately 60–70% of the movement cycle, corresponding to the propulsion phase following shock absorption. The dominant muscles were the LGM and PL in SLDJ, and the LGM, PL, BF, and ST in DLDJ.

**Figure 5:**
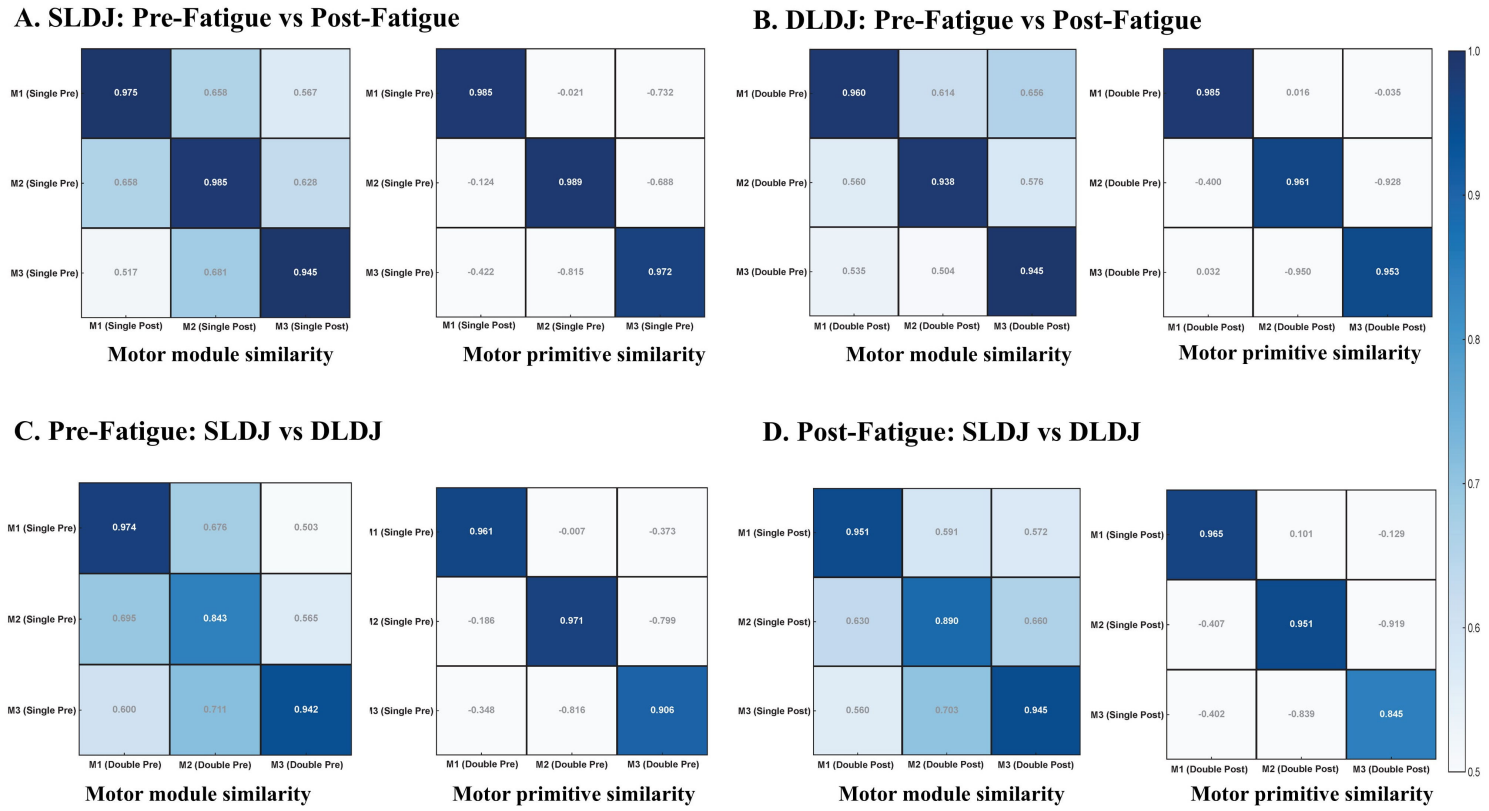
Muscle synergy similarity. Similarity of corresponding muscle synergy modules across drop jump tasks and fatigue conditions. Similarity was assessed using both scalar product and Pearson correlation coefficients for muscle weighting patterns and temporal activation profiles. Values greater than 0.80 indicate high similarity between corresponding modules.

**Figure 5** shows the similarity of corresponding synergies between SLDJ and DLDJ, as well as within each task between pre- and post-fatigue conditions, for both motor modules and motor primitives. The results showed consistently high similarity across all comparisons, with both scalar product and pearson correlation coefficients exceeding 0.80.

### 3.4 Muscle contributions and temporal characteristics

**Figure 6:**
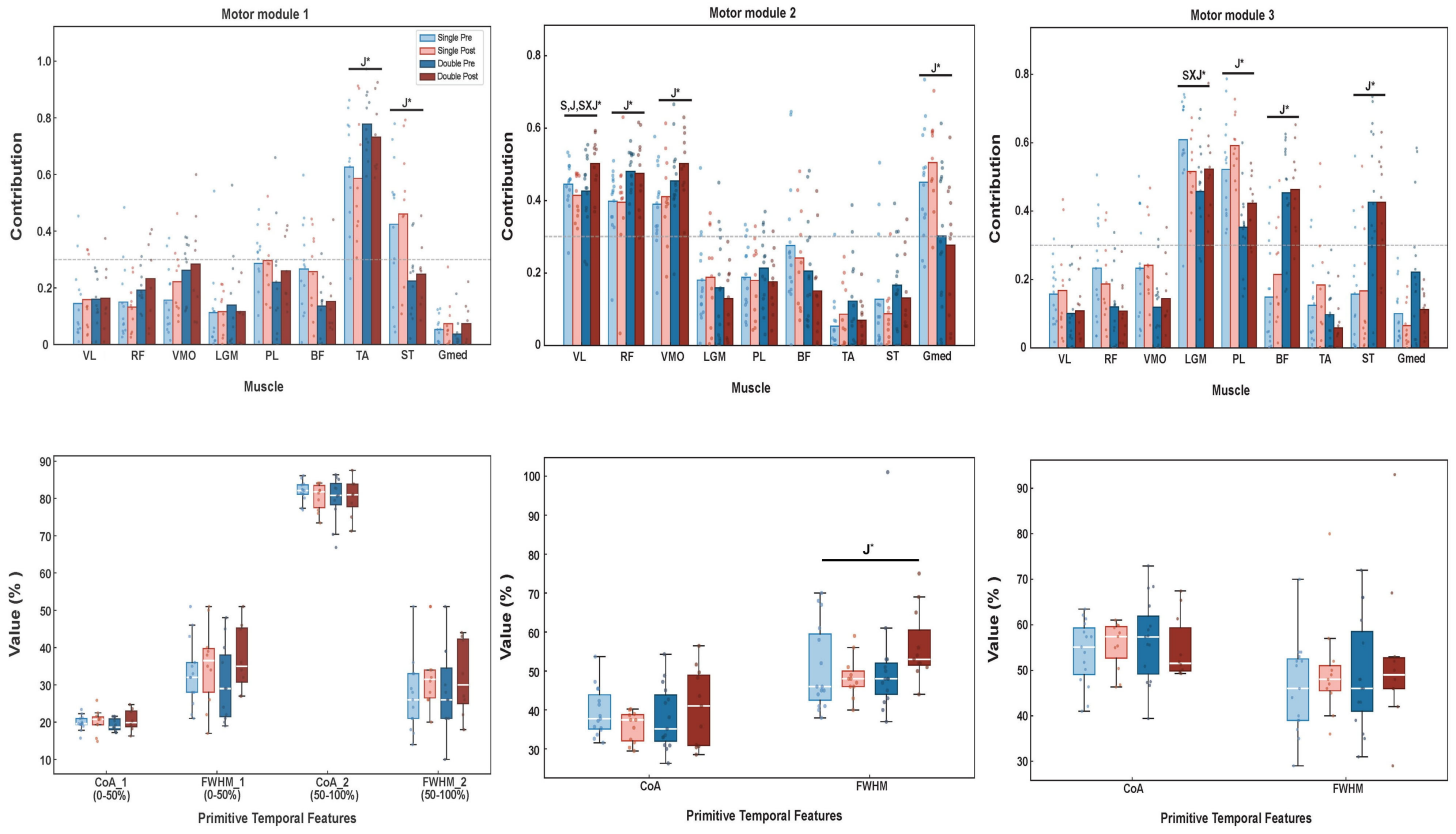
Muscle contributions and temporal characteristic. Bar plots represent the relative muscle contributions within homologous synergy modules across fatigue conditions and DJ types, with weighting coefficients greater than 0.30 indicating dominant contributors. Box plots represent temporal features, including the CoA and full width at FWHM. J*, S*, and S × J* indicate significant main effects of jump type, fatigue, and their interaction, respectively. **(A)** Single-leg drop jump. **(B)** Double-leg drop jump.

**Figure 6** shows the statistical analysis results of muscle contributions and temporal characteristics. For Synergy 1, significant main effects of jump type were observed for the TA(t = −2.08, p = 0.03, ηp²=0.1, 95%CI=-0.20, -0.01) and ST(t = 2.26, p = 0.02, ηp²=0.11, 95%CI=0.02, 0.28). Compared with SLDJ, DLDJ consistently exhibited greater TA contribution and lower ST contribution, regardless of fatigue condition. No significant differences were found for temporal features.

For Synergy 2, significant main effects of jump type were identified for the VL (t = −2.57, *p* = 0.01, ηp²=0.12, 95%CI=-0.15, -0.02), RF (t = −2.32, *p* = 0.02, ηp²=0.1, 95%CI=-0.13, -0.01), VMO (t = −2.00, *p* = 0.04, ηp²=0.07, 95%CI=-0.18, -0.002), and Gmed (t = 3.22, *p*<0.01, ηp²=0.17, 95%CI=0.09, 0.37). Compared with SLDJ, DLDJ consistently showed greater contributions from RF and VMO, and lower contribution from Gmed. In addition, VL demonstrated a significant main effect of fatigue (t = −2.40, *p* = 0.02, ηp²=0.1, 95%CI=-0.14, -0.01) as well as a significant interaction effect (t = 2.40, *p* = 0.02, ηp²=0.1, 95%CI=0.02, 0.19). Although simple effects analysis did not reach statistical significance, a trend was observed whereby VL contribution decreased following fatigue in SLDJ(0.446 ± 0.068 to 0.415 ± 0.061) but increased in DLDJ(0.426 ± 0.101 to 0.502 ± 0.076). Regarding temporal features, a significant main effect of jump type was found for FWHM (t = −1.97, *p* = 0.04, ηp²=0.07, 95%CI=-0.288, -0.001), with DLDJ exhibiting longer FWHM compared with SLDJ under both fatigue conditions.

For Synergy 3, significant main effects of jump type were observed for the PL (t = 3.57, *p* < 0.01, ηp²=0.21, 95%CI=0.08, 0.26), BF (t = −3.97, *p* < 0.01, ηp²=0.25, 95%CI=-0.34, -0.11), and ST (t = 2.78, *p* < 0.01, ηp²=0.14, 95%CI=-0.38, -0.07). Compared with SLDJ, DLDJ consistently showed greater contributions from BF and ST, and lower contribution from PL. Furthermore, a significant interaction effect was observed for the LGM (t = 2.08, *p* = 0.03, ηp²=0.08, 95%CI=0.01, 0.31). Simple effects analysis revealed that LGM contribution significantly decreased after fatigue during SLDJ (0.609 ± 0.131 to 0.516 ± 0.103; t = 3.8, *p* = 0.01, d=1.14, 95%CI=0.05, 0.18), whereas no significant change was observed in DLDJ(0.458 ± 0.163 to 0.523 ± 0.126). No significant differences were found in temporal features for Synergy 3.

## 4 Discussion

This study used NMF based muscle synergy analysis to compare lower-limb muscle synergy characteristics between SLDJ and DLDJ in female athletes pre and post fatigue. The findings partially supported our hypotheses. The number and overall structure of muscle synergies remained stable across fatigue conditions and DJ types, suggesting that the CNS maintained a consistent modular control strategy. Adaptations to fatigue and task demands were mainly reflected in changes in muscle weighting and activation features. Moreover, the significant interaction effects in specific muscle contributions indicate that fatigue related modulation of muscle synergies was task specific. It should also be noted that both SLDJ and DLDJ showed decreased RSI and prolonged GCT after fatigue. Since RSI is a sensitive indicator of SSC efficiency and lower-limb neuromuscular output, its reduction further confirmed the validity of the fatigue protocol and supported the interpretation of the muscle synergy results (Fitzpatrick et al., 2019).

### 4.1 Muscle synergies between SLDJ and DLDJ

The number of extracted muscle synergies is generally considered to reflect the dimensionality of CNS motor control (d’Avella et al., 2003). In this study, three synergy modules were extracted in both SLDJ and DLDJ before and after fatigue, with high similarity between corresponding modules. This suggests that the overall neuromuscular control structure was preserved across fatigue conditions and DJ types, supporting the view that muscle synergies represent relatively stable functional units within the CNS (Bizzi and Cheung, 2013).

However, significant jump-type effects on muscle contributions and temporal features indicate task-specific modulation within these stable modules. Module 1 was active around IC and TO. Compared with DLDJ, SLDJ additionally recruited ST as a dominant contributor, suggesting greater demand for knee stabilization during the initial and final phases of the movement. Module 2 corresponded to the eccentric braking phase. In this module, DLDJ relied more on quadriceps contributions, whereas SLDJ showed greater dependence on Gmed and a shorter FWHM. This pattern indicates different neuromuscular control strategies between the two DJ tasks and suggests that SLDJ may require greater proximal control during landing. FWHM represents the duration of the activation pattern (Janshen et al., 2021), and its shortening has previously been reported to co-occur with altered lower limb mechanical loading (Munoz-Martel et al., 2021). Based on this association, one possible hypothesis is that the shorter FWHM observed in SLDJ reflects a more compressed, proximally weighted control strategy during landing; however, because GRF and joint-level kinetics were not directly measured in this study, this interpretation should be regarded as hypothesis-generating rather than a confirmed mechanism, and warrants direct verification through simultaneous kinematic-kinetic analysis in future work. Module 3 corresponded to the propulsion phase. During this phase, DLDJ showed significantly greater contributions from BF and ST, together with LGM and PL, indicating a hip–ankle coordinated propulsion strategy. In contrast, SLDJ relied more heavily on ankle-related muscles. This more distal-dominant propulsion pattern may partly explain why a fatigue related reduction in JH was observed only during SLDJ.

### 4.2 Effects of fatigue on muscle synergies and modulation by jump type

SLDJ and DLDJ showed highly similar muscle synergy patterns pre and post fatigue, consistent with previous findings from running and repetitive upper-limb tasks showing that fatigue does not markedly alter overall synergy structure (Thomas et al., 2023; Xu et al., 2025; Li and He, 2026). Unlike these repetitive movements, DJ is a short-duration explosive task in which muscle activation may rely more on pre-planned control and allow limited real-time adjustment. Thus, the present findings may further support the robustness of muscle synergies under fatigue and across different movement tasks (Ortega-Auriol et al., 2018).

The main finding of this study was that fatigue induced task-specific modulation of muscle contributions across different DJ types. In Module 2, VL contribution showed opposite fatigue-related trends between SLDJ and DLDJ, decreasing after fatigue in SLDJ but increasing in DLDJ. In SLDJ, the reduced VL contribution may reflect decreased reliance on the quadriceps under fatigue. Given that this module also involved Gmed, the increased Gmed weighting after fatigue suggests that SLDJ may rely more on proximal muscle contributions to help maintain single-leg stability under fatigued conditions. In contrast, the increased VL contribution in DLDJ may represent a compensatory adjustment to preserve landing absorption during the more stable bilateral landing task. In Module 3, LGM contribution significantly decreased after fatigue during SLDJ, whereas no significant change was observed during DLDJ. As LGM plays an important role in ankle elastic energy storage and release during the SSC, its reduced contribution may directly affect propulsive output (Komi, 2000; Patti et al., 2025). Combined with the ankle-dominant propulsion pattern observed in SLDJ, the decrease in LGM contribution may further explain the post-fatigue reduction in JH during SLDJ. In contrast, during DLDJ, the combined contribution of BF and ST within the same module may have reduced the influence of fatigue on ankle-related muscle contribution, thereby helping to maintain LGM contribution.

Overall, these findings suggest that fatigue does not uniformly affect muscle synergies, but selectively modulates muscle weighting according to task demands, with SLDJ showing greater neuromuscular sensitivity to fatigue than DLDJ.

### 4.3 Practical implications

These findings carry implications for athlete neuromuscular assessment and training. The demonstration that SLDJ is more sensitive to fatigue induced neuromuscular changes suggests it may serve as a more sensitive functional screening tool for detecting neuromuscular deficits under fatigued conditions. From a training perspective, athletes in sports that frequently involve movements similar to SLDJ, such as basketball and volleyball, may benefit from targeted strengthening or neuromuscular training of the LGM and Gmed to help maintain jump performance under fatigue and neuromuscular control under fatigue in athletes.

### 4.4 Limitation

Several limitations should be acknowledged. First, although the sample size was determined using an *a priori* power analysis and was comparable to that of many similar studies (Hu et al., 2025; Xu et al., 2025), the *post hoc* power calculated from the observed effect sizes ranged from 0.71 to 0.86. This suggests that some analyses may still have been underpowered. In addition, all participants were female basketball and volleyball athletes, which limits the generalizability of the findings to other populations. Second, as this was an exploratory study, kinematic and kinetic variables were not collected simultaneously. This limits the depth of interpretation regarding the potential mechanisms underlying the observed muscle synergy changes. Third, fatigue was assessed primarily using indirect indicators, including jump performance, heart rate, and perceived exertion. Because objective physiological measures such as blood lactate concentration or maximal voluntary contraction loss were not collected, the physiological state of fatigue could not be directly confirmed. Forth, EMG recordings were limited to the dominant limb to ensure comparability with SLDJ. Thus, DLDJ results reflect dominant limb neuromuscular control only and do not fully represent bilateral coordination. Future studies should include bilateral EMG recordings to better characterize neuromuscular control during DLDJ.

## 5 Conclusion

This study showed that SLDJ and DLDJ shared stable lower-limb muscle synergy structures before and after fatigue, suggesting that the CNS maintained a consistent modular control strategy across DJ tasks. Task differences and fatigue effects were mainly reflected in changes in specific muscle contributions and temporal features, rather than in synergy number or overall structure. Fatigue selectively modulated muscle contributions in a task specific manner, with SLDJ showing greater neuromuscular sensitivity than DLDJ, suggesting that it may serve as a more sensitive functional screening task for detecting fatigue-related neuromuscular control alterations.

## Data Availability

The raw data supporting the conclusions of this article will be made available by the authors, without undue reservation.

## References

[B1] Anderson T. Wasserman E. B. Shultz S. J. (2019). Anterior cruciate ligament injury risk by season period and competition segment: An analysis of national collegiate athletic association injury surveillance data. J. Athl Train 54, 787–795. doi: 10.4085/1062-6050-501-17 31322904 PMC6709760

[B2] Bizzi E. Cheung V. C. K. (2013). The neural origin of muscle synergies. Front. Comput. Neurosci. 7, 51. doi: 10.3389/fncom.2013.00051 23641212 PMC3638124

[B3] Bizzi E. Cheung V. C. K. d’Avella A. Saltiel P. Tresch M. (2008). Combining modules for movement. Brain Res. Rev. 57, 125–133. doi: 10.1016/j.brainresrev.2007.08.004 18029291 PMC4295773

[B4] Borg E. Borg G. Larsson K. Letzter M. Sundblad B.-M. (2010). An index for breathlessness and leg fatigue. Scand. J. Med. Sci. Sports 20, 644–650. doi: 10.1111/j.1600-0838.2009.00985.x 19602182

[B5] Ch Y. Pv L. Jc G. (2011). An investigation of lower extremity energy dissipation strategies during single-leg and double-leg landing based on sagittal and frontal plane biomechanics. Hum. Mov. Sci. 30, 624–35. doi: 10.1016/j.humov.2010.11.010 21411162

[B6] Chappell J. D. Herman D. C. Knight B. S. Kirkendall D. T. Garrett W. E. Yu B. (2005). Effect of fatigue on knee kinetics and kinematics in stop-jump tasks. Am. J. Sports Med. 33, 1022–1029. doi: 10.1177/0363546504273047 15983125

[B7] Chen Y. Yang C. Côté J. N. (2024). Few sex-specific effects of fatigue on muscle synergies in a repetitive pointing task. J. Biomech. 163, 111905. doi: 10.1016/j.jbiomech.2023.111905 38183760

[B8] d’Avella A. Bizzi E. (2005). Shared and specific muscle synergies in natural motor behaviors. Proc. Natl. Acad. Sci. U.S.A. 102, 3076–3081. doi: 10.1073/pnas.0500199102 15708969 PMC549495

[B9] d’Avella A. Portone A. Fernandez L. Lacquaniti F. (2006). Control of fast-reaching movements by muscle synergy combinations. J. Neurosci. 26, 7791–7810. doi: 10.1523/JNEUROSCI.0830-06.2006 16870725 PMC6674215

[B10] d’Avella A. Saltiel P. Bizzi E. (2003). Combinations of muscle synergies in the construction of a natural motor behavior. Nat. Neurosci. 6, 300–308. doi: 10.1038/nn1010 12563264

[B11] Fitzpatrick J. F. Akenhead R. Russell M. Hicks K. M. Hayes P. R. (2019). Sensitivity and reproducibility of a fatigue response in elite youth football players. Sci. Med. Football 3, 214. doi: 10.1080/24733938.2019.1571685 37339054

[B12] Fort-Vanmeerhaeghe A. Bishop C. Montalvo A. M. Buscà B. Arboix-Alió J. (2023). Effects of exercise-induced neuromuscular fatigue on jump performance and lower-limb asymmetries in youth female team sport athletes. J. Hum. Kinet. 89, 19–31. doi: 10.5114/jhk/174073 38053949 PMC10694723

[B13] Franceschi A. Robinson M. A. Owens D. Brownlee T. Ferrari Bravo D. Enright K. (2023). Reliability and sensitivity to change of post-match physical performance measures in elite youth soccer players. Front. Sports Act. Living 5. doi: 10.3389/fspor.2023.1173621 37521097 PMC10374287

[B14] Hajiloo B. Anbarian M. Esmaeili H. Mirzapour M. (2020). The effects of fatigue on synergy of selected lower limb muscles during running. J. Biomech. 103, 109692. doi: 10.1016/j.jbiomech.2020.109692 32151383

[B15] Hermens H. J. Freriks B. Disselhorst-Klug C. Rau G. (2000). Development of recommendations for SEMG sensors and sensor placement procedures. J. Electromyogr Kinesiol 10, 361–374. doi: 10.1016/s1050-6411(00)00027-4 11018445

[B16] Herzog M. Krafft F. C. Fiedler J. Berger D. J. Sloot L. H. d’Avella A. . (2025). The central nervous system adjusts muscle synergy structure and tightly controls rollator-supported transitions between sitting and standing. J. NeuroEng. Rehabil. 22, 96. doi: 10.1186/s12984-025-01622-y 40281643 PMC12032710

[B17] Hirashima M. Oya T. (2016). How does the brain solve muscle redundancy? Filling the gap between optimization and muscle synergy hypotheses. Neurosci. Res. 104, 80–87. doi: 10.1016/j.neures.2015.12.008 26724372

[B18] Hu C. Du N. Li Y. Girard O. Mei T. (2025). Enhancing jump performance through blood flow restriction squat exercise: A muscle synergy analysis using wavelet packet transformation. J. Sports Sci. Med. 24, 578–588. doi: 10.52082/jssm.2025.578 40933326 PMC12418193

[B19] Ivanenko Y. P. Cappellini G. Dominici N. Poppele R. E. Lacquaniti F. (2005). Coordination of locomotion with voluntary movements in humans. J. Neurosci. 25, 7238–7253. doi: 10.1523/JNEUROSCI.1327-05.2005 16079406 PMC6725226

[B20] Janshen L. Santuz A. Arampatzis A. (2021). Muscle synergies in patients with multiple sclerosis reveal demand-specific alterations in the modular organization of locomotion. Front. Hum. Neurosci. 14. doi: 10.3389/fnhum.2020.593365 33584221 PMC7873056

[B21] Kamitani A. Hara K. Fujii Y. Yoshida S. (2023). Landing posture in elite female athletes during a drop vertical jump before and after a high-intensity ergometer fatigue protocol: A study of 20 Japanese women’s soccer league players. Orthop. J. Sports Med. 11, 23259671231171859. doi: 10.1177/23259671231171859 37435587 PMC10331781

[B22] Komi P. V. (2000). Stretch-shortening cycle: A powerful model to study normal and fatigued muscle. J. Biomech. 33, 1197–1206. doi: 10.1016/s0021-9290(00)00064-6 10899328

[B23] Kotsifaki R. Sideris V. King E. Bahr R. Whiteley R. (2023). Performance and symmetry measures during vertical jump testing at return to sport after ACL reconstruction. Br. J. Sports Med. 57, 1304–1310. doi: 10.1136/bjsports-2022-106588 37263763

[B24] Li Z. He K. (2026). Effects of fatigue on the activation characteristics and synergistic patterns of lower limb muscles during running. Front. Physiol. 17, 1741432. doi: 10.3389/fphys.2026.1741432 41867248 PMC12999458

[B25] Liu Y. Liu R. Wan X. Chen C. Wang Y. Yu W. . (2024). The effect of short-term kinesiology taping on neuromuscular controls in hallux valgus during gait: A study of muscle and kinematic synergy. IEEE Trans. Neural Syst. Rehabil. Eng. 32, 3199–3209. doi: 10.1109/TNSRE.2024.3451651 39208038

[B26] Liu C. Peng W. Qu W. Zhang Z. Sun J. He J. . (2025). Gender differences in the impact of fatigue on lower limb landing biomechanics and their association with anterior cruciate ligament (ACL) injuries: A systematic review and meta-analysis. PloS One 20, e0321925. doi: 10.1371/journal.pone.0321925 40334188 PMC12058186

[B27] Merrigan J. J. O’Toole K. B. Wutzke C. J. Jones M. T. (2022). Kinetic and kinematic analysis of various drop jump performances in army reserve officer training corps cadets. J. Strength Conditioning Res. 36, 738. doi: 10.1519/JSC.0000000000004041 34132221

[B28] Munoz-Martel V. Santuz A. Bohm S. Arampatzis A. (2021). Proactive modulation in the spatiotemporal structure of muscle synergies minimizes reactive responses in perturbed landings. Front. Bioeng. Biotechnol. 9. doi: 10.3389/fbioe.2021.761766 34976964 PMC8716881

[B29] Ortega-Auriol P. A. Besier T. F. Byblow W. D. McMorland A. J. C. (2018). Fatigue influences the recruitment, but not structure, of muscle synergies. Front. Hum. Neurosci. 12, 217. doi: 10.3389/fnhum.2018.00217 29977197 PMC6021531

[B30] Ortega-Auriol P. Besier T. McMorland A. J. C. (2025). Effect of surface electromyography normalisation methods over gait muscle synergies. J. Electromyography Kinesiology 80, 102968. doi: 10.1016/j.jelekin.2024.102968 39721229

[B31] Pappas E. Hagins M. Sheikhzadeh A. Nordin M. Rose D. (2007). Biomechanical differences between unilateral and bilateral landings from a jump: Gender differences. Clin. J. Sport Med. 17, 263–268. doi: 10.1097/JSM.0b013e31811f415b 17620779

[B32] Patti A. Thomas E. Giustino V. Rossi C. Paoli A. Drid P. . (2025). Effects of foam rolling and static stretching on ankle dorsiflexion and jumping ability: A randomized controlled trial. Biol. Sport 42, 163–170. doi: 10.5114/biolsport.2025.150042 41048241 PMC12490304

[B33] Pedley J. S. Lloyd R. S. Read P. J. Moore I. S. Myer G. D. Oliver J. L. (2025). Drop jump vertical kinetics identify male youth soccer players at greater risk of non-contact knee injury. Phys. Ther. Sport 73, 48–56. doi: 10.1016/j.ptsp.2025.03.003 40073652 PMC13244297

[B34] Prieske O. Demps M. Lesinski M. Granacher U. (2017). Combined effects of fatigue and surface instability on jump biomechanics in elite athletes. Int. J. Sports Med. 38, 781–790. doi: 10.1055/s-0043-111894 28768338

[B35] Saito H. Yokoyama H. Sasaki A. Nakazawa K. (2023). Muscle synergy patterns as altered coordination strategies in individuals with chronic low back pain: A cross-sectional study. J. NeuroEngineering Rehabil. 20, 69. doi: 10.1186/s12984-023-01190-z 37259142 PMC10230697

[B36] Santuz A. Ekizos A. Janshen L. Baltzopoulos V. Arampatzis A. (2017). On the methodological implications of extracting muscle synergies from human locomotion. Int. J. Neural Syst. 27, 1750007. doi: 10.1142/S0129065717500071 27873551

[B37] Selya A. S. Rose J. S. Dierker L. C. Hedeker D. Mermelstein R. J. (2012). A practical guide to calculating Cohen’s f(2), a measure of local effect size, from PROC MIXED. Front. Psychol. 3, 111. doi: 10.3389/fpsyg.2012.00111 22529829 PMC3328081

[B38] Shourijeh M. S. Flaxman T. E. Benoit D. L. (2016). An approach for improving repeatability and reliability of non-negative matrix factorization for muscle synergy analysis. J. Electromyogr Kinesiol 26, 36–43. doi: 10.1016/j.jelekin.2015.12.001 26755163

[B39] Stojanović E. Stojiljković N. Scanlan A. T. Dalbo V. J. Berkelmans D. M. Milanović Z. (2018). The activity demands and physiological responses encountered during basketball match-play: A systematic review. Sports Med. 48, 111–135. doi: 10.1007/s40279-017-0794-z 29039018

[B40] Taylor J. B. Ford K. R. Nguyen A.-D. Shultz S. J. (2016). Biomechanical comparison of single- and double-leg jump landings in the sagittal and frontal plane. Orthop. J. Sports Med. 4, 2325967116655158. doi: 10.1177/2325967116655158 27482527 PMC4954550

[B41] Thomas S. J. Castillo G. C. Topley M. Paul R. W. (2023). The effects of fatigue on muscle synergies in the shoulders of baseball players. Sports Health 15, 282–289. doi: 10.1177/19417381221084982 35492023 PMC9950986

[B42] Turpin N. A. Uriac S. Dalleau G. (2021). How to improve the muscle synergy analysis methodology? Eur. J. Appl. Physiol. 121, 1009–1025. doi: 10.1007/s00421-021-04604-9 33496848

[B43] van Melick N. Meddeler B. M. Hoogeboom T. J. Nijhuis-van der Sanden M. W. G. van Cingel R. E. H. (2017). How to determine leg dominance: The agreement between self-reported and observed performance in healthy adults. PloS One 12, e0189876. doi: 10.1371/journal.pone.0189876 29287067 PMC5747428

[B44] Wilder J. N. Riggins E. R. Noble R. A. Lelito C. M. Widenhoefer T. L. Almonroeder T. G. (2021). The effects of drop vertical jump technique on landing and jumping kinetics and jump performance. J. Electromyogr Kinesiol 56, 102504. doi: 10.1016/j.jelekin.2020.102504 33242751

[B45] Xu Y. Yang Y. He S. Yang C. Zhang S. Fu W. . (2025). Running-induced fatigue influences lower extremity muscle synergy and related biomechanics. Gait Posture 119, 163–170. doi: 10.1016/j.gaitpost.2025.03.008 40117855

[B46] Yeow C. H. Lee P. V. S. Goh J. C. H. (2010). Sagittal knee joint kinematics and energetics in response to different landing heights and techniques. Knee 17, 127–131. doi: 10.1016/j.knee.2009.07.015 19720537

[B47] Zhang Q. Li F. Trowell D. A. Hou M. Qiu Z. Chen S. . (2025). Optimizing stretch-shortening cycle performance: Effects of drop height and landing strategy on lower-limb biomechanics in drop jumps. PeerJ 13, e19490. doi: 10.7717/peerj.19490 40416624 PMC12103163

[B48] Zou L. Zhang X. Jiang Z. Wu X. Zhang Q. (2024). Influences of fatigue and anticipation on female soccer players’ biomechanical characteristics during 180° pivot turn: Implication for risk and prevention of anterior cruciate ligament injury. Front. Physiol. 15, 1424092. doi: 10.3389/fphys.2024.1424092 39282087 PMC11394182

